# The spatial representation of loudness in a timbre discrimination task

**DOI:** 10.1177/20416695231213213

**Published:** 2023-11-14

**Authors:** Sarah Koch, Torsten Schubert, Sven Blankenberger

**Affiliations:** Department of Psychology, Martin-Luther-Universität Halle-Wittenberg, Halle, Germany

**Keywords:** SMARC effect, ATOM, cognition, audition, polarity correspondence principle

## Abstract

When participants decide whether a presented tone is loud or soft they react faster to loud tones with a top-sided response key in comparison to a bottom-sided response key and vice versa for soft tones. This effect is comparable to the well-established horizontal Spatial-Numerical Association of Response Codes (SNARC) effect and is often referred to as Spatial-Musical Association of Response Codes (SMARC) effect for loudness. The SMARC effect for loudness is typically explained by the assumption of a spatial representation or by the polarity correspondence principle. Crucially, both theories differ in the prediction of the SMARC effect when loudness is task-irrelevant. Therefore, we investigated whether the SMARC effect still occurs in a timbre discrimination task: Participants (*N* = 36) heard a single tone and classified its timbre with vertically arranged response keys. Additionally, the tone's loudness level varied in six levels. In case of a spatial representation, the SMARC effect should still occur while in case of polarity corresponding principle, the effect should be absent. Results showed that the SMARC effect was still present and that the differences between top-sided and bottom-sided responses were a linear function of loudness level indicating a continuous spatial representation of loudness.

Several studies indicated that magnitude dimensions might be spatially represented. This close link between space and magnitude was first investigated in the domain of numbers, leading to the observation of the Spatial-Numerical Association of Response Codes effect (SNARC; Dehaene et al., 1993). In that study, participants classified numbers as odd or even by pressing a left-sided or right-sided response key. Reaction times were shorter when participants responded with a left-sided response key to small numbers compared to a right-sided response key and vice versa for large numbers. The SNARC effect is usually explained by a spatial representation of numbers in the form of a mental number line with small numbers represented on the left and large numbers represented on the right (Dehaene et al., 1993; Feigenson et al., 2004; Restle, 1970). This spatial representation is assumed to interfere with the spatial code of the motor responses (Keus & Schwarz, 2005). In recent decades, SNARC-like effects were found for other magnitude dimensions (for a review, see Macnamara et al., 2018) as well as for auditory dimensions like pitch (Lega et al., 2020; Lidji et al., 2007; Rusconi et al., 2006) and loudness (Fairhurst & Deroy, 2017; Hartmann & Mast, 2017). Due to the wide range of various dimensions with spatial associations, the general term Spatial-Association of Response Codes effect (SARC; Macnamara et al., 2018) was proposed to refer to the effect for any of these dimensions.

Despite numerous studies investigating the SARC effect for different dimensions, little is known whether the general SARC effect derives from the same origin as the SNARC effect, for example, a spatial representation of the respective magnitude. This is especially the case for dimensions which do not bear a magnitude information in explicit manner as is the case for auditory dimensions like pitch and loudness. This article aims to investigate the occurrence of the SARC effect for loudness, to which we will refer as Spatial-Musical Association of Response Codes (SMARC) effect. This term was originally introduced to refer to spatial associations for pitch by Rusconi et al. (2006) but has been extended to loudness by Bruzzi et al. (2017).

The occurrence of the SMARC effect is usually explained by two different theoretical approaches: the polarity correspondence principle (Chang & Cho, 2015; Proctor & Cho, 2006) and the assumption of a generalized magnitude representation system as described in A Theory of Magnitude, short ATOM (Bueti & Walsh, 2009; Walsh, 2003, 2015). According to the polarity correspondence principle, the SMARC effect occurs due to a match between the polarity codes of the response and the stimulus dimension. These polarity codes are assumed to be categorical with a positive and negative polarity and formed in binary classification tasks for both the response and the stimulus dimension. Right-sided responses are coded as “+ polarity” while left-sided responses are coded as “− polarity” (Cho et al., 2012; Proctor & Cho, 2006). In case of a continuous stimulus dimension, such as loudness, polarity correspondence principle implies that the dimension must dichotomized into two distinct categories. The polarity codes are formed either due to an external reference point or due to task demands (Cho et al., 2012). With regard to loudness, loud and soft tones are assumed to have a “+ polarity” and a “− polarity,” respectively (Chang & Cho, 2015). If participants have to respond to a loud tone with a right-sided response key, the polarity codes of the response and the stimulus dimension are compatible and, therefore, participants respond faster. Likewise, when the polarity codes of the two dimensions do not match, as it is the case when reacting with the left-sided response key to a loud tone, participants are slower.

This prediction was investigated in a study of Chang and Cho (2015) with two different experiments. Participants either judged the loudness of a second tone (the probe tone) relative to the first tone (the reference tone) or they judged the timbre of the second tone. Crucially, there was still a reference tone in the timbre discrimination task serving as external reference point so that the continuous loudness dimension could still be dichotomized. A significant interaction between loudness level and response side was found in both tasks indicating the occurrence of the SMARC effect. However, to test the polarity correspondence principle as an explanation for the SMARC effect, it is crucial to investigate the occurrence of the effect when loudness is not relevant for the task and no reference point is given as done in a study by Cho et al. (2012). The authors investigated the SARC effect for pitch and assumed that “if the SMARC effect is based on categorical codes for pitch height, no SMARC effect would be obtained under conditions in which such categorical codes of the target tone pitch are not formed” (Cho et al., 2012, p. 727). They investigated this assumption in a series of different binary classification tasks. In the pitch discrimination tasks, participants had either to decide whether a second tone was higher or lower than a first tone or they had to decide whether a single tone was high or low. In the timbre discrimination tasks, participants had to classify a tone with regard to its timbre. The tone was either presented alone or was preceded by a reference tone. When pitch was the task-relevant dimension, a SARC effect for pitch was observed independently of whether or not a reference tone was presented, probably due to task demands. In contrast, when participants had to judge the timbre of the tone, the SARC effect for pitch was only present when a reference tone was presented. The authors concluded that “nonmusicians need a context, provided by the referent tone, which causes the irrelevant pitch height to be coded” (Cho et al., 2012, p. 733).

In contrast to the polarity correspondence principle, other authors explain the SMARC effect with the assumption that loudness might be represented as magnitude in the context of ATOM (Bruzzi et al., 2017; Hartmann & Mast, 2017). According to ATOM, space, time, and quantity are represented on a common metric (Bueti & Walsh, 2009; Walsh, 2003, 2015). Due to this shared representation, Walsh (2003) concluded that every magnitude dimension in ATOM should show a SARC effect (SQUARC effect for Spatial-Quantity Association of Response Codes), with faster left-sided responses to small magnitudes compared to right-sided responses and vice versa for large magnitudes. As already mentioned, this pattern was found for various magnitude dimensions (Macnamara et al., 2018). The assumption of spatial associations for various magnitude dimensions was further developed to the assumption that ATOM-related magnitudes might be spatially organized on a mental magnitude line (Holmes & Lourenco, 2011) comparable to the idea of a mental number line (Dehaene et al., 1993). We will refer to this assumption of a spatial representation as second account to explain the SMARC effect.

However, to explain the SMARC effect by ATOM or in general by the assumption of a spatially organized magnitude representation, one has to assume that loudness is represented as a magnitude. Although loudness is often referred to as auditory magnitude (Fairhurst & Deroy, 2017; Ren et al., 2011), the dimension was not included in the theoretical conceptualization of ATOM (Bueti & Walsh, 2009; Walsh, 2003). Nevertheless, loudness fulfills one of ATOM's core assumptions, namely being a quantitative dimension in contrast to a qualitative dimension (Stevens & Galanter, 1957). Furthermore, loudness interacts with numerical value as well as physical size, two dimensions which are considered to be part of ATOM (Alards-Tomalin et al., 2015; Hartmann & Mast, 2017; Heinemann et al., 2013; Takeshima & Gyoba, 2013). Altogether, this is consistent with the assumption that loudness might be represented as magnitude according to ATOM.

It is important to note that some studies, which interpreted the SMARC effect in terms of ATOM investigated the SMARC effect only in the vertical dimension (Bruzzi et al., 2017; Fernandez-Prieto et al., 2017). For example, in the study of Bruzzi et al. (2017), participants had to judge whether a probe tone was louder or softer than the reference tone by pressing a top-sided or bottom-sided response key. A SMARC effect occurred, which indicated that loud tones are represented top and soft tones are represented bottom. One might argue that a vertical representation is not in line with the assumption of a horizontal spatial representation of magnitude. However, neither the spatial associations by ATOM nor the assumed spatial representation of magnitudes are explicitly restricted to the horizontal dimension. Furthermore, several other ATOM-related magnitudes seem to have a vertical representation (for a review regarding numbers, see Winter et al., 2015). With regard to the SMARC effect, neither the vertical nor the horizontal dimension seem to be a “preferred” axis. Although the horizontal SMARC effect was absent in some earlier studies (Ren et al., 2011), it was found by later studies (Chang & Cho, 2015; Fairhurst & Deroy, 2017; Hartmann & Mast, 2017). The same is true for the vertical SMARC effect, which was found in most studies (Bruzzi et al., 2017; Fernandez-Prieto et al., 2017) but not in the study of Fairhurst and Deroy (2017). To summarize, the vertical direction of a SMARC effect does not necessarily contradict a spatial representation as explanation.

Although not explicitly mentioned in the context of the SMARC effect, other explanations can also be considered. One theoretical approach aims to explain the SNARC effect by the assumption of a spatial organization of stimuli in the working memory due to task demands (van Dijck & Fias, 2011; Gevers et al., 2003). However, because there is no empirical evidence for the working memory account as explanation for the SMARC effect we will focus on the distinction between the polarity correspondence principle and the assumption of a spatial representation as possible explanations.

The SMARC effect as observed in prior studies was previously explained by the assumption of a spatial representation of loudness or by the polarity correspondence principle. Crucially, the methods of previous studies allow for both interpretations. Most studies, which investigated the SMARC effect, either conducted a loudness discrimination task (Bruzzi et al., 2017; Fairhurst & Deroy, 2017; Fernandez-Prieto et al., 2017; Hartmann & Mast, 2017) or presented a reference tone in the timbre judgment task (Chang & Cho, 2015) and, therefore, the creation of polarity codes for the stimulus dimension can not be excluded. Additionally, most studies only presented one loud tone and one soft tone so that the auditory material was already dichotomized (e.g., Chang & Cho, 2015). However, the results from these studies are also in line with the assumption of a spatial representation of loudness and due to its quantitative nature, loudness could be also part of ATOM.

The results from prior studies lead to the question whether the SMARC effect still occurs when loudness is not relevant for the task and no external reference point is given. Therefore, in the current study, we investigated the occurrence of the SMARC effect in a timbre discrimination task without a reference tone. Participants heard one single tone and classified the tone with regard to its timbre with a top-sided or a bottom-sided response key. We chose the vertical dimension instead of the horizontal dimension due to the lack of studies investigating the SMARC effect in a timbre discrimination task in the vertical dimension. Furthermore, we used a real vertical arrangement of response keys, that is, the top-sided response key was spatially above the bottom-sided response key as used by other studies (Bruzzi et al., 2017; Fernandez-Prieto et al., 2017). Some authors claim, that arrangements in which the response keys are near and far to the subject are comparable to a real vertical arrangement in the context of spatial compatibility effects (e.g., Rusconi et al., 2006). However, the distinction is important in the context of this study. In an arrangement with near and far response keys, a potential SMARC effect might be explained by the magnitude association between loud and large distance as well as soft and small spatial distance.^1^ In contrast, in a real vertical arrangement, the spatial distance to the subject is the same for both response keys.

Crucially, the assumption of a spatial representation and the polarity correspondence principle differ in the prediction of a SMARC effect when loudness is irrelevant for the task and when no reference tone is presented. According to the polarity correspondence principle, loudness must be coded into two polarities, so that stimulus and response codes can match and a SMARC effect can occur (Proctor & Cho, 2006). In line with the results from Cho et al. (2012), we assume that this dichotomization can either occur due to task demands or due to an external reference point such as a reference tone (Cho et al., 2012). Both are not given in the current study and therefore, the coding of loudness into two distinct polarities seems unlikely. In contrast, the assumption of a spatially organized magnitude representation for loudness predicts that the SMARC effect should be present in a timbre discrimination task even without a reference tone. Due to the intrinsic spatial representation of loudness, the mere presentation of a tone with a specific loudness level should lead to an activation of the corresponding spatial information. This in turn should lead to the occurrence of interference effects, which would occur due to compatible or incompatible relations between the spatial representation of loudness and the response codes, comparable as for the SNARC effect (Keus & Schwarz, 2005).

It might be, that participants are able to form an internal reference point as it seems to be the case with pitch for professional musicians (Cho et al., 2012). However, in this case, the two accounts would predict different patterns for the SMARC effect. The polarity correspondence principle implies a categorical SMARC effect, that is, the difference between reaction times for top-sided and bottom-sided responses is roughly the same for all loudness levels with the same polarity. Contrary, a continuous spatial representation would lead to a SMARC effect with larger differences for extreme stimulus values, comparable to the SNARC effect (Dehaene et al., 1993).

Because loudness is a quantitative dimension (Stevens & Galanter, 1957) and due to its interaction with other magnitudes (Alards-Tomalin et al., 2015; Hartmann & Mast, 2017; Heinemann et al., 2013; Takeshima & Gyoba, 2013) we assume that loudness is represented as a magnitude and that the SMARC effect is due to a spatial representation. Therefore, we hypothesized that the effect would still be present in a timbre discrimination task without a reference tone. If, in contrast, the SMARC effect originates from the match of polarity codes no effect should occur due to the missing external reference point.

## Methods

The study's design as well as the statistical analyses were preregistered on aspredicted.org (https://aspredicted.org/8KV_4FB).

### Participants

*N* = 37 healthy students of Martin Luther University Halle-Wittenberg participated for course credit. Age was restricted to a range between 18 and 35 years to ensure, that participants had no age-related decline in their hearing ability. Furthermore, no professional musicians (i.e., people who study or have studied music or work fulltime or part-time as musician) were allowed to participate in the study. The mean age was *M* = 22.86 years (*SD* = 3.1). 30 participants reported having a female gender. All participants reported being right-handed, that they had either no vision impairments or used a correction in the experiment, and that they had no hearing impairments. 31 participants reported playing or have played an instrument for more than a year. All participants gave written informed consent to participate. The study was conducted in accordance with the declaration of Helsinki and has been approved by the ethics committee of the DGPs (Deutsche Gesellschaft für Psychologie). The planned sample size was *N* = 36 based on a power analysis for a one-tailed one-sample *t*-test with α = .05, power 1 − β = .9 and an effect size of *d* = −0.5. After we reached our planned sample size, we checked the mean error rates for each participant and excluded one participant due to an overall error rate exceeding 15%. According to our preregistration, we collected data from one additional participant. Therefore, *N* = 36 participants were included in the final data analysis.

### Material

The auditory stimuli consisted of six sawtooth wave tones and six rectangle wave tones. For each timbre, six different sound pressure levels ranging from approximately 40–65 dBA in 5 dBA steps were realized. All tones had a frequency of 440 Hz. Tones were generated with Audacity (Version 3.1.0; The Audacity Team, 2019) with a sampling rate of 48 kHz and presented via headphones (Sennheiser HD471). Each sound file had a duration of 800 ms. Participants responded to the tones by pressing a top-sided or a bottom-sided response key; both keys were vertically aligned on a response box with the bottom-sided response key and top-sided response key being approximately 1 and 16 cm above the table surface, respectively. Stimulus presentation and data recording were realized with PsychoPy (Version 2021.2.3; Peirce et al., 2019).

### Design and Procedure

The study followed a 6 × 2 × 2 within-subjects design with the following factors, sound pressure level (6 levels, from 40 to 65 dBA), timbre (2 levels, a sawtooth wave tone or a rectangle wave tone), and response mapping (2 levels, top-sided response key for sawtooth wave tones and bottom-sided response key for rectangle wave tones and vice versa), which resulted, altogether, in 24 within-subjects conditions.

Each trial started with the presentation of a fixation cross in the middle of the screen for 500 ms. After a fixed foreperiod of 500 ms, the tone was presented for a maximum of 800 ms or until participants made a response. There was no time window for responses, that is participants could still react after the tone had ended. After participants made their response, an inter-trial interval (ITI) with a random, uniform-distributed duration between 1 and 2 s was presented before the next trial started. Participants were instructed to classify the presented tone as sawtooth wave tone or rectangle wave tone as fast and correct as possible by pressing a top-sided or bottom-sided response key. They were not told that the tones could vary in loudness but in order to make them familiar with the auditory material they heard all tones during instruction phase.

Response mapping varied between two sessions. Half of the participants responded in the first session with the top-sided response key to the sawtooth wave tone and with the bottom-sided response key to the rectangle wave tone and vice versa in the second session. The other half of the participants had the opposite order of response mapping. Additionally, key-hand mapping was varied between participants. Half of the participants pressed the top-sided response key with their right thumb and the bottom-sided response key with their left thumb, the other half had the opposite arrangement. Participants were randomly assigned to one of the four condition groups.

In both sessions, each tone was presented 56 times, resulting in 672 experimental trials in each session. Each session consisted of 14 blocks à 48 experimental trials plus 3 warm-up trials at the beginning of a block. Additionally, participants performed 48 training trials at the beginning of each session in which they received feedback about their reaction time and correctness of their response. Both sessions took approximately 50 min.

### Data Analyses

We used R (Version 4.1.2; R Core Team, 2021) and the R-packages *ez* (Version 4.4; Lawrence, 2016), *papaja* (Version 0.1.1; Aust & Barth, 2020), and *tidyverse* (Version 1.3.1; Wickham et al., 2019) for all our analyses. Reaction times from incorrect trials (7.5%) were discarded from further analyses. We calculated the trimmed mean reaction times with a trimming amount of 20% (Rosenberger & Gasko, 1983) and the mean error rate for each participant and within-subject condition. Additionally, we calculated the difference of reaction times ( $dRT=RT_{top}-RT_{bottom}$ ) for each participant, loudness level, and timbre (Fias et al., 1996).

In a first step, we conducted a mixed 6 × 2 × 2 × 2 × 2 ANOVA with sound pressure level, timbre, and response side as within-subject factors as well as order of response mapping, and key-hand mapping as between-subject factors. In case of violations against the assumption of sphericity, corrected *p*-values according to the Greenhouse–Geisser adjustment (GG) will be reported. For the effect sizes in the ANOVA analyses, we will report generalized eta-squared $\eta_{G}^{2}$ because it allows comparisons of effect sizes across studies and its reporting is recommended to facilitate cumulative statistics (Bakeman, 2005; Lakens, 2013).

## Results

**Figure 1.**
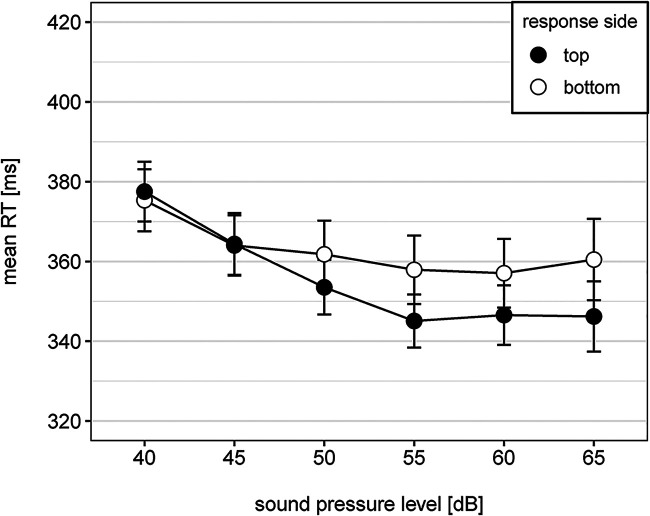
Mean reaction time as a function of sound pressure level and response side. Note. Error bars represent 95% within CI (Morey, 2008).

Overall, reaction time decreased with increasing loudness level, as can be seen in Figure 1. The main effect of loudness was significant, F(5,160)=39.47 , MSE=344.54 , p<.001 (GG), ${\hat{\eta }}_{G}^{2}=.024$ . Furthermore, participants responded faster when they made a top-sided response compared to a bottom-sided response (356 vs. 363 ms), F(1,32)=9.38 , MSE=1,201.77 , p=.004 , ${\hat{\eta }}_{G}^{2}=.004$ and participants who pressed the top-sided response key with their left thumb where faster compared to participants who pressed the top-sided response key with their right thumb (338 vs. 380 ms), F(1,32)=5.55 , MSE=70,815.15 , p=.025 , ${\hat{\eta }}_{G}^{2}=.122$ . Reaction time differences between both timbres were small (360 ms for the rectangle wave tone vs. 358 ms for the sawtooth wave tone) and did not differ significantly, F(1,32)=0.94 , MSE=1,476.04 , p=.338 , ${\hat{\eta }}_{G}^{2}<.001$ .

The SMARC effect is indicated by the interaction between sound pressure level and response side, which is shown in Figure 1. Descriptively, participants responded faster to loud tones when pressing a top-sided response key compared to pressing a bottom-sided response key but for the soft tones there was only a small difference in reaction times between both response sides. Nevertheless, the interaction was significant, F(5,160)=8.97 , MSE=191.63 , p<.001 (GG), ${\hat{\eta }}_{G}^{2}=.003$ . Because we later analyzed the SMARC effect in a linear regression analysis, we refrained from testing the differences between reaction times for the bottom-sided and top-sided response key via post hoc tests at this point.

**Figure 2.**
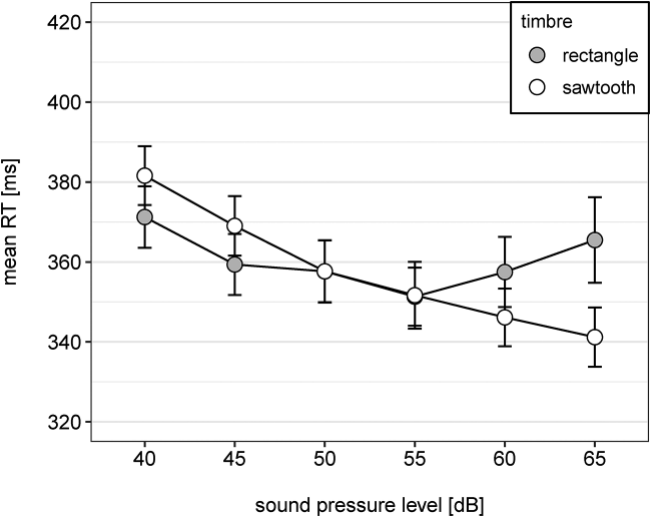
Mean reaction time as a function of sound pressure level and timbre. Note. Error bars represent 95% within CI (Morey, 2008).

There was also a significant interaction between loudness level and timbre, F(5,160)=11.83 , MSE=535.83 , p<.001 (GG), ${\hat{\eta }}_{G}^{2}=.011$ which is illustrated in Figure 2. For the sawtooth wave tone, reaction time decreased with increasing sound pressure level. For the rectangle wave tone, reaction time first decreased with increasing sound pressure level and then increased for the loudest tones.

There was also a significant interaction between response side and key-hand mapping, F(1,32)=18.76 , MSE=1,201.77 , p<.001 , ${\hat{\eta }}_{G}^{2}=.008$ . Participants who pressed the top-sided response key with their right thumb were faster when they responded with the top-sided response key compared to the bottom-sided response key (372 vs. 389 ms). This difference was smaller and reversed for participants who pressed the top-sided response key with their left thumb (339 vs. 336 ms). Additionally, the three-way interaction between order of mapping, timbre, and response side was significant, F(1,32)=30.83 , MSE=8,328.03 , p<.001 , ${\hat{\eta }}_{G}^{2}=.083$ . All other interactions were non-significant (*p* > .07).

**Figure 3.**
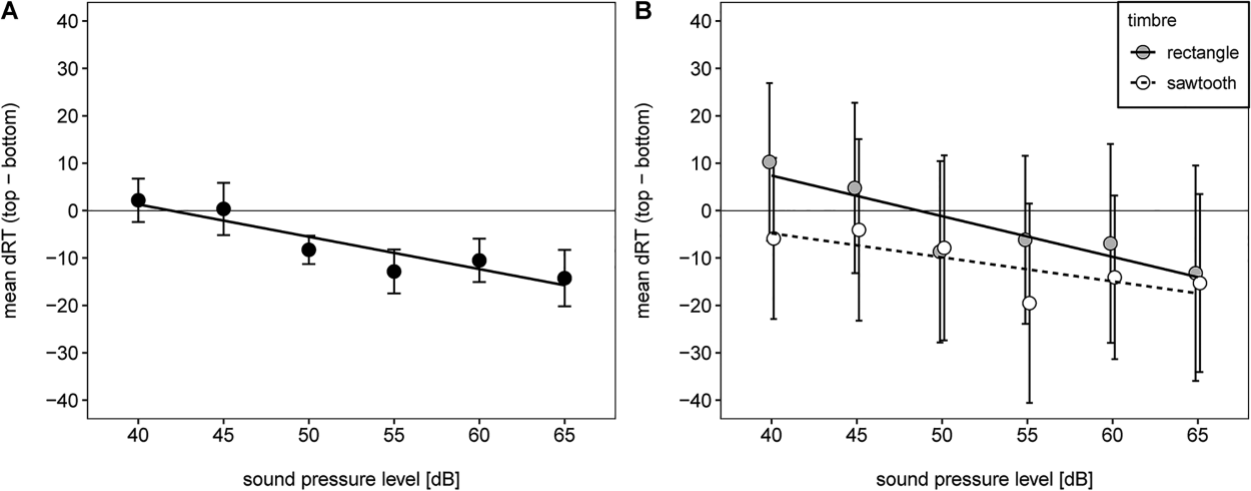
Mean dRT and regression line as a function of sound pressure level for the overall data (A) and separate for each timbre (B). Note. Error bars represent 95% within CI (Morey, 2008).

For a more detailed analysis of the SMARC effect, we ran a linear regression analysis with sound pressure level as predictor and mean dRT as dependent variable separately for each participant. The resulting regression coefficients were then tested against zero via a one-tailed (preregistered) one-sample *t*-test (Fias et al., 1996; Lorch & Myers, 1990). According to our preregistration procedure, we estimated and tested the linear regression coefficients for the overall mean dRT and separately for each timbre due to the significant interaction between sound pressure level and timbre. Mean dRT as a function of sound pressure level is illustrated in Figure 3. For each timbre, mean dRT decreased with increasing sound pressure level. Mean regression coefficients were significantly smaller than zero in all three analyses, $M_{overall}=-0.68$ , 95% CI [−∞,−0.42] , t(35)=−4.38 , p<.001 , $M_{sawtooth}=-0.51$ , 95% CI [−∞,−0.24] , t(35)=−3.26 , p=.001 and $M_{rectangle}=-0.86$ , 95% CI [−∞,−0.47] , t(35)=−3.78 , p<.001 . A two-tailed, paired *t*-test showed that there was no significant difference between the regression coefficients for sawtooth wave tone and rectangle wave tone, $M_{D}=0.35$ , 95% CI [−0.12,0.82] , t(35)=1.50 , p=.142 .

The results from the ANOVA as well as from the linear regression analysis indicate an interaction between response side and sound pressure level. However, participants might have used the tone of the previous trial as a reference to dichotomize the loudness of the tone in the ongoing trial. In order to investigate the influence of the loudness level of the preceding trial, we categorized each trial as having a higher, lower, or the same sound pressure level compared to the tone of the previous trial.^2^ Then, we calculated the trimmed mean (trimming amount = 20%) of reaction times for each participant, sound pressure level, response side, timbre, and loudness change. If the SMARC effect were mainly due to the relative loudness change, participants should be always faster with the top-sided response key when responding to a louder tone compared to when responding with the bottom-sided response key irrespective of its total loudness level. Please note, that in our experimental design, there is an unequal number of observations for each sound pressure level and loudness change combination because we did not control the probability of being a louder or softer tone for each sound pressure level. Furthermore, the loudest tone could never be softer and the softest tone could never be louder compared to previous trial. Due to these missing observations, we relied on a descriptive investigation of the influence of loudness change.

**Figure 4.**
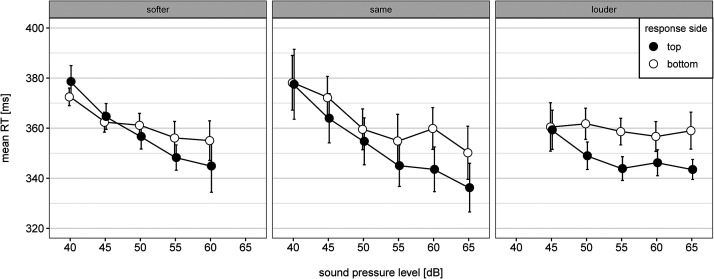
Mean RT as a function of sound pressure level, response side, and loudness change relative to the tone's loudness of the previous trial. Note. Error bars represent 95% within CI (Morey, 2008).

As can be seen in Figure 4, roughly similar interactions between sound pressure level and response side emerged for all three loudness changes. While the relative loudness change seemed to influence the asymmetry of the interaction pattern, it did not change the overall effect and the interaction patterns mirrored the overall interaction effect visualized in Figure 1.

## Discussion

The aim of the current study was to investigate whether the vertical SMARC effect for loudness occurs in a timbre discrimination task, that is, when loudness is not relevant for the task. Indeed, we found a significant interaction between response side and sound pressure level which was supported by the results from the linear regression analysis: For loud tones, participants responded faster with a top-sided response key in comparison to a bottom-sided response key. In contrast, there was no difference between reaction times for the soft tones. Despite this asymmetry, the results from the linear regression analysis indicate that the difference of reaction time was a linear function of sound pressure level. The results are in line with our hypothesis that the vertical SMARC effect still occurs in a timbre discrimination task.

A comparable asymmetric interaction was also found in other studies investigating the horizontal SMARC effect (Chang & Cho, 2015; Hartmann & Mast, 2017) and might be due to a perceptual and neural processing bias for loud tones (Neuhoff, 1998; Puigcerver et al., 2019). Furthermore, loud sounds or sounds with increasing loudness indicate higher proximity to a potential threat and are therefore probably more likely to trigger a response (Puigcerver et al., 2019). This could be an explanation, why the difference between the different response sides is more pronounced for loud tones. Another possible explanation is the main effect of response side with overall faster reactions for the top-sided responses. This was not the case in other studies investigating the SMARC effect in the vertical dimension in which the difference of reaction times were comparable for loud and soft tones (e.g., Bruzzi et al., 2017).

We counterbalanced the assignment between response side and responding hand. Crucially, key-response mapping did not interact with the SMARC effect, that is, the three-way interaction between response side, sound pressure level, and key-hand mapping was not significant. This indicates that the SMARC effect is due to a spatial association between the vertical response side and sound pressure level and is independent from the spatial features of the effectors comparable to the SNARC effect (Dehaene et al., 1993).

The observation of the SMARC effect in a timbre discrimination task without a reference tone is in line with the assumption of a spatial representation of loudness but not in line with the polarity correspondence principle. There was neither an external reference tone nor had participants to categorize loudness due to task demands. Therefore, it seems unlikely that loudness was coded into two distinct polarities.

It might be possible that participants created an internal reference point as it was found for professional musicians with the SARC effect for pitch (Cho et al., 2012). However, there is no empirical evidence supporting this assumption for non-musicians with regard to loudness. Furthermore, even in case of using the average loudness as internal reference point, the SMARC effect would have been categorical, with the same negative dRTs for all loud tones and the same positive dRTs for all soft tones. Instead, dRT was a linear function of sound pressure level indicating a continuous spatial representation. Additionally, the SMARC effect was mainly driven by the absolute loudness level of the tones and not by the loudness change which would be the case, if participants would have used the loudness level of the previous trial as reference point. However, because we did not systematically control for loudness change, we relied on a descriptive analysis. Furthermore, the relative loudness change seemed to have an influence on the symmetry of the interaction. Future studies should therefore address this issue and entangle the influence of loudness change on the one hand and the absolute sound pressure level on the other hand.

In brief, while polarity correspondence principle can not be fully ruled out as an explanation, the results are more in line with the assumption of a spatial representation of loudness. According to this assumption, the processing of loudness results in an activation of the corresponding spatial position on a spatial representation, which then interferes with the spatial position of the response keys (Dehaene et al., 1993; Holmes & Lourenco, 2011; Keus & Schwarz, 2005).

The question remains whether this spatial representation is due to the representation of loudness as a magnitude in terms of ATOM (Walsh, 2003) as suggested in other studies (Bruzzi et al., 2017; Hartmann & Mast, 2017). As already mentioned by other authors, SARC effects cannot be interpreted as magnitude indicator and the spatial representation of stimuli might also rely on the concept of order (Casasanto & Pitt, 2019; Pitt & Casasanto, 2022) or spatial organization of stimuli in the working memory due to task demands (van Dijck & Fias, 2011; Gevers et al., 2003). Could the SMARC effect found in the current study be explained by these alternative explanations? Due to the fact that, in the present study, loudness was not relevant to the task it seems implausible that a spatial arrangement of loudness was built and maintained as a primary representation for task processing in working memory. Nevertheless, the effect could still occur assuming that loudness is associated with a specific order information. The design of the study does not allow us to disentangle order information from magnitude information and, therefore, the question remains open.

However, besides these theoretical considerations, there are several other empirical findings beyond the SMARC effect, which indicate that loudness is represented as magnitude dimension. Loudness is a quantitative dimension (Stevens & Galanter, 1957), which is one criterion for being considered as ATOM-related magnitude (Walsh, 2003, 2015). Furthermore, loudness interacts with ATOM-related magnitude dimensions like numbers (Alards-Tomalin et al., 2015; Hartmann & Mast, 2017; Heinemann et al., 2013) or object size (Takeshima & Gyoba, 2013). Hartmann and Mast (2017) found that participants respond faster to numerically large numbers in a loud-spoken voice compared to a soft-spoken voice and vice versa for small numbers. This was the case either when participants had to judge the loudness or had to judge the numerical value. The authors also investigated the occurrence of both the SNARC effect and the SMARC effect and their possible interrelation. The authors found that both effects exclusively occurred, when the associated dimension was task-relevant. Furthermore, although a loudness-magnitude interaction was observed in both tasks, the interaction did not interact with neither the SNARC effect in the number discrimination task nor the SMARC effect in the loudness discrimination task. Therefore, the authors concluded that magnitude interactions and spatial associations might originate from different mechanisms. This interpretation would be in line with the assumption that loudness is processed as a magnitude but that the SMARC effect relies on a spatial representation probably due to its order information (Pitt & Casasanto, 2022).

The direction of the vertical SMARC effect, as observed in the current study, is in line with the vertical spatial representation of the ATOM-related magnitude numbers. Small numbers are assumed to be represented bottom while large numbers are represented top (Aleotti et al., 2022; Schwarz & Keus, 2004; Wiemers et al., 2017; Winter et al., 2015). This direction of a spatial association for magnitudes might be due to the natural association between verticality and quantity in our environment (Lakoff & Johnson, 2003; Winter et al., 2015). In general, an increasing amount of something is typically accompanied by an increasing level in verticality, for example, the rising of water level when a glass is filled. However, although neither ATOM nor the assumption of spatially magnitude representations do explicitly narrow the spatial representation to the horizontal dimension, it remains unclear how vertical and horizontal representations of magnitude dimensions might relate to each other.

We found a significant interaction between timbre and sound pressure level, which might indicate that both timbres were processed differently on different loudness levels, which might influence the SMARC effect. However, there was no significant difference between the two regression coefficients and they were both significantly smaller than zero, indicating the occurrence of the SMARC effect for both timbres. The interaction between timbre and sound pressure level in the current study resembles the results from Melara and Marks (1990) investigating crossmodal correspondences between different auditory dimensions. In their third experiment, the authors varied the loudness level and duty cycle of rectangle wave tones the latter leading to a “twangy” and a “hollow” sound impression. Loud “twangy” sounds as well as soft “hollow” sounds lead to a reaction time benefit compared to “twangy” soft and loud “hollow” sounds. This is comparable to faster reaction times for loud sawtooth wave tones and soft rectangle wave tones compared to the reversed combination of loudness and sound timbre as we observed in this study. Whether crossmodal correspondences between loudness and timbre exist and whether they can have an influence on spatial-loudness associations, as it is the case for pitch (Pitteri et al., 2017, 2021) needs further investigation.

In sum, our study showed that the vertical SMARC effect is present even when loudness is not task relevant, which speaks against the polarity correspondence principle as explanation for the observed effect. Instead, the current findings are consistent with the assumption that the effect is due to a continuous spatial representation of loudness which interacts with the spatial code of the vertical aligned motor responses. This spatial representation might be due to a magnitude representation of loudness in terms of ATOM but further empirical validation of this claim is needed. The investigation of interdependences between the SMARC effect and SARC effects of ATOM-related magnitudes like numbers as done by Hartmann and Mast (2017) could help to understand whether the effects rely on the same representation system. Additionally, further research is required to understand how the vertical and horizontal representation of loudness relate to each other and whether they form a common mental magnitude space.

## Notes

1. We thank one anonymous reviewer for pointing this out.

2. We thank Nathan Van der Stoep for suggesting this additional analysis.
